# Electrochemical Determination of Nitroguanidine among Other Explosives Using Polymerized 2‑Nitrophenol/Reduced Graphene Oxide-Modified Glassy Carbon Electrode

**DOI:** 10.1021/acsomega.5c10881

**Published:** 2026-02-20

**Authors:** Şener Sağlam

**Affiliations:** Engineering Faculty, Chemistry Department, Istanbul University−Cerrahpaşa, Avcılar, Istanbul 34320, Türkiye

## Abstract

In this study, a novel sensor working electrode was fabricated by functionalizing a glassy carbon (GC) electrode surface with electrochemically reduced graphene oxide (ERGO) and poly-2-nitrophenol (P2NP, electrochemically polymerized, used as a hydrogen-bonding substrate) using the proposed square-wave voltammetry (SWV) method toward the highly selective and sensitive electrochemical sensing of nitroguanidine (NG), a friction- and impact-insensitive high explosive increasingly used in energetic formulations, even in the presence of other types of energetic materials. NG showed a characteristic reduction peak at approximately −1.27 V. A linear response was obtained between 0.5 and 100 mg L^–1^ (4.80 × 10^–6^–9.61 × 10^–4^ mol L^–1^), and the limit of detection (LOD) was found as 0.12 mg L^–1^ (1.15 × 10^–6^ mol L^–1^). The developed GC/ERGO/P2NP sensor electrode successfully determines NG in both synthetic and real energetic material mixtures (20-fold) with high recovery rates. The sensor maintained its high selectivity (20 mg L^–1^ NG) even with the addition of potential soil interferents at a 50-fold excess (10-fold for Fe^3+^, Pb^2+^, and Cu^2+^) including Cl^–^, SO_4_^2–^, NO_2_^–^, NO_3_^–^, NH_4_^+^, K^+^, Na^+^, Ca^2+^, and Mg^2+^, and electroactive camouflage materials (10-fold) such as paracetamol, caffeine, acetylsalicylic acid, aspartame, d-glucose, and detergent. Finally, clay soil samples contaminated with NG were analyzed using the proposed method, and the results were statistically compared with LC–MS using Student’s *t-*test and *F-*test, confirming its reliability for real-world environmental monitoring.

## Graphical abstract

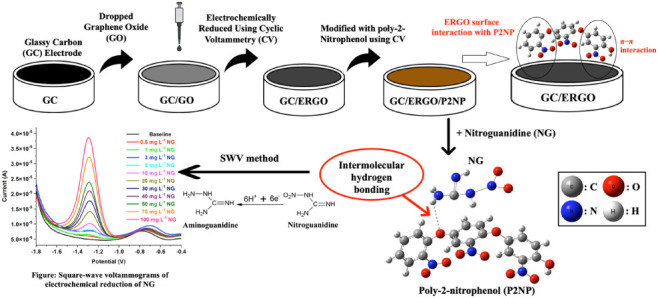

## 1 Introduction

Nitroguanidine (NG) is a high-nitrogen compound that functions as a new insensitive explosive, resistant to friction and impact.^1^ With the chemical formula CH_4_N_4_O_2_, this colorless crystalline material is frequently used, especially in military applications,^2–4^ because of its low sensitivity and thermal stability. NG has been employed in artillery shell propellants, offering advantages such as reducing barrel erosion, lowering the flame temperature, and stabilizing recoil balance.^5,6^ Additionally, it is used in smokeless pyrotechnic compositions, gas generators for automobile airbag systems, and rocket propellants, and regrettably, it has also been associated with terrorist acts.^2,7,8^ NG exhibits high polarity, resulting in significant solubility in water while remaining insoluble in most organic solvents.^9,10^ Due to its high aqueous solubility, it can persist in environments such as soil and groundwater for extended periods, consequently posing serious threats to aquatic organisms, algae, and fish.^11–13^ Under exposure to visible and ultraviolet light, NG’s toxicity increases as its photodegradation product, cyanoguanidine, may decompose into hydrogen cyanide under acidic conditions. NG can enter the environment through explosion residues and potential leaks from NG production facilities.^14–16^ Due to its inadequate degradation by soil microorganisms and high solubility in water, it may ultimately enter the human food chain.^17–19^ Regarding safety standards, while no official occupational exposure limits have been established, the U.S. EPA lists a chronic oral Reference Dose (RfD) of 0.1 mg/kg/day to safeguard against systemic toxicity.^20^

NG exhibits high environmental persistence and poses significant challenges in analytical detection. Thus, the development of sensitive and specific analytical methods is necessary to precisely evaluate their possible environmental impact and set up efficient monitoring strategies. Over the past several years, scientists have developed various sensitive analytical techniques to detect and quantify trace amounts of NG in environmental samples, such as volumetry,^21^ colorimetry,^8^ liquid chromatography–mass spectrometry (LC–MS),^22–24^ high-performance thin-layer chromatography (HPTLC),^25^ UV–vis spectroscopy,^26^ and fluorescence spectroscopy.^27^ Even though chromatographic and spectroscopic methods provide adequate sensitivity and selectivity, they have several drawbacks, such as expensive expenses, difficult sample preparation, matrix effect susceptibility, high solvent requirements, and prolonged analysis periods.^28,29^ On the other hand, electrochemical techniques provide a few significant benefits, including affordable equipment, quick reaction, extensive linear range, excellent sensitivity and specificity, compactness, and precise on-site analysis.^30–37^

Graphene oxide (GO) is a suitable material for electrode modification in electrochemical sensors owing to its large specific surface area, the presence of functional groups enabling selective interactions, tunable surface chemistry, and favorable electron transfer properties. In addition, GO can be easily converted to chemically and/or electrochemically reduced GO (ERGO), thus significantly increasing its electrical conductivity and enabling much more sensitive analysis of the analyte.^38,39^ Apart from carbon-based nanomaterials, conductive polymer films formed by electropolymerization have attracted significant attention in sensor fabrication. Electropolymerization offers distinct advantages, such as the formation of uniform and stable films directly on the electrode surface with a controllable thickness. Among various monomers, 2-nitrophenol is of particular interest. Poly-2-nitrophenol (P2NP) films possess a rich surface chemistry containing hydroxyl (−OH) and nitro (−NO_2_) groups. These functional groups can form specific supramolecular interactions, particularly hydrogen bonds, with analytes bearing amino groups. Therefore, P2NP serves as an excellent modification agent to enhance selectivity and sensitivity through the effective preconcentration of the target molecules on the electrode surface.^37^ There are many studies in the literature on the electrochemical determination of energetic substances. Most of these studies have focused on highly sensitive military-grade explosives. However, due to their risk of uncontrolled reactions, these explosives present significant disadvantages in terms of impact sensitivity, as well as safe storage and transportation.^40^ However, in recent years, insensitive explosives have increasingly replaced highly sensitive explosives, as they eliminate the associated disadvantages. The use of these explosives has significant economic and logistical implications for storage, handling, and transportation. Additionally, this class of explosives reduces the possibility of mass detonations and allows for high-density storage in confined spaces or warehouses where munitions are stored close together.^9,41^ To date, only one study has been reported in the literature concerning the electrochemical determination of NG. Alassane Moussa et al. prepared an electrode modification with a solution containing multiwalled carbon nanotubes (MWCNTs) and polyvinylpyrrolidone (PVP) on the GC electrode surface, and the sensor exhibited a linear response toward NG in the range of 3 to 100 mg L^–1^ (2.88 × 10^–5^–9.61 × 10^–4^ mol L^–1^) using the SWV technique.^42^

NG poses an environmental risk due to its potential release through explosive residues and production facility leaks. Its poor biodegradability and high solubility in water may facilitate its migration into the food chain. Accordingly, its selective and direct determination in the presence of other energetic compounds is vital for forensic investigations, environmental monitoring, and homeland security. In this study, a novel electrochemical sensor and SWV method were proposed for the specific and sensitive determination of NG, an insensitive high explosive, in the presence of different types of energetic substances. To improve sensing capability, a composite electrode surface (this specific combination has not been reported before for the selective detection of NG) was designed by combining electrochemically reduced graphene oxide (ERGO, which provides enhanced conductivity and surface area) and poly-2-nitrophenol (P2NP), enabling effective hydrogen bonding interactions with NG, thereby facilitating efficient hydrogen bond formation with NG molecules. The electrochemical impedance spectroscopy (EIS) and cyclic voltammetry (CV) were utilized to characterize the developed sensor working electrode. In addition, the sensor was tested in the presence of different types of energetic compounds (binary and multicomponent mixtures), typical soil ions, and electrochemically active camouflage materials. The proposed method was statistically compared with the LC–MS method^23^ using statistical *t*- and *F*-tests, based on analyses conducted with NG-contaminated clay soil samples.

## 2 Experimental Section

### 2.1 Chemicals, Solutions, and Instruments

Different types of energetic materials, such as 2,4,6-trinitrotoluene (TNT), 2,4-dinitrotoluene (DNT), 2,4,6-trinitrophenylmethylnitramine (tetryl), 1,3,5-trinitro-1,3,5-triazacyclohexane (RDX), octahydro-1,3,5,7-tetranitro-1,3,5,7-tetrazosine (HMX), 3-nitro-1,2,4-triazol-5-one (NTO), 3-(nitrooxy) nitrate-2,2-bis­[(nitrooxy)­methyl]­propyl (PETN), Octol (containing 70% HMX and 30% TNT), and Comp. B (containing 60% RDX, 39% TNT, and 1% wax), used for sensitive and selective electrochemical analysis of NG, were supplied by Machinery & Chemistry Industries Institution Chemical (MKEK) of Türkiye. Lithium perchlorate (LiClO_4_, 99.99% trace metals basis), sodium chloride (NaCl, 99.0%), sodium phosphate monobasic dihydrate (NaH_2_PO_4_·2H_2_O, ≥98%), sodium phosphate dibasic dihydrate (Na_2_HPO_4_·2H_2_O, ≥98%), tetrabutylammonium bromide (TBABr, ≥98%), and tetrabutylammonium tetrafluoroborate (TBATBF_4_, ≥99%) were used as supporting electrolytes to increase the electrical conductivity of the measurement solution in the determination of NG. Alumina slurry (Baikowski International 0.05 mm, Baikalox, 0.05 CR), technical alcohol, and acetone were utilized for the surface pretreatment of the electrodes. 2-nitrophenol (2NP, ≥98%) and graphene oxide (GO, powder, 15–20 sheets, 4–10% edge-oxidized) were used in the modification of the working electrode, and all remaining chemicals used for GC electrode modification were supplied by Sigma-Aldrich.

The stock solution of each energetic substance (TNT, DNT, Tetryl, RDX, HMX, NTO, Octol, and Comp. B) were prepared at 1000 mg L^–1^ by dissolving in extra pure acetone (ACN). Ammonium nitrate (NH_4_NO_3_, ≥98%), camouflage materials that have colors and appearances comparable to NG (detergent, d-glucose, caffeine (≥98%), paracetamol (≥98%), aspartame (≥98%), acetylsalicylic acid (≥99%)), and common soil ions were prepared at 1000 mg L^–1^ in 0.1 mol L^–1^ phosphate buffer (pH 7). The electrochemical impedance measurements (EIS) and cyclic voltammetry (CV) measurements were performed using a 5 mmol L^–1^ [Fe­(CN)_6_]^3–/4–^ solution prepared in 0.1 mol L^–1^ HCl with 0.1 mol L^–1^ KCl used as the supporting electrolyte. The possibility of hydrogen bonding interactions was further supported by Density Functional Theory (DFT) calculations.

A Metrohm Autolab Potentiostat/Galvanostat (PGSTAT204, Netherlands) was used for the voltammetric experiments. The platinum electrode (Pt), the Ag/AgCl electrode (containing 3 mol L^–1^ NaCl), and the glassy carbon electrode (GCE, MF-2012) (BASi stationary voltammetry electrodes; 1.6 mm, surface area of 0.02 cm^2^) were used as auxiliary, reference, and working electrodes. The Fourier Transform Infrared Spectrometer (FTIR) IRTracer-100, Shimadzu, was used to confirm the electrochemical reduction of GO to ERGO (screen-printed electrode, SPE, Metrohm C11L). Note that Screen-Printed Electrodes (SPEs) were utilized as the substrate for FTIR analysis. This was necessary because the physical dimensions of the GCE (BASi MF-2012, approximately 7.5 cm in length) were incompatible with the measurement compartment of the Shimadzu IRTracer-100 instrument. Raman spectroscopy measurements were performed using an i-Raman Plus 532 H system. X-ray photoelectron spectroscopy (XPS) analyses were performed using a Thermo Scientific K-Alpha instrument. SEM-EDX measurements were carried out with a field-emission scanning electron microscope (FE-SEM; FEI Quanta 450 FEG, Hillsboro, OR, USA), whereas X-ray diffraction (XRD) analyses were conducted with a Malvern Panalytical/Empyrean Multicore Pix 3D third generation diffractometer. Transmission Electron Microscopy (TEM) images of GO were obtained using an FEI TALOS F200S TEM operated at 200 kV. Atomic Force Microscopy (AFM) analysis was performed with XE-100 AFM from Park Systems. The Shimadzu 8040 LC–MS liquid chromatograph–mass spectrometer was used to statistically validate the suggested electrochemical method with the LC–MS method^23^ in clay soil samples contaminated with NG.

### 2.2 Method Optimization

For the optimization of the developed SWV method, parameters such as working electrode selection, selection of the measurement medium and supporting electrolyte, selection of the measurement pH, and selection of the amount of 2-NP monomer used in the electrode modification were investigated. Since certain energetic compounds are soluble in acetone (ACN), ACN is added to the measurement medium to allow for the electrochemical analysis of NG in the presence of these materials.

### 2.3 Preparation of the Modified Working Electrode

Before measurements, the GC electrode serving as the working electrode in the electrochemical determination of the NG was cleaned. This cleaning procedure is carried out as follows: after several minutes of polishing with an alumina suspension in circular motions, the GC working electrode was thoroughly rinsed with distilled water. This cleaning protocol was deemed adequate, as no peak was observed in the baseline solution (blank) during the SWV measurement.^43^

After the cleaning protocol, 10 μL (the maximum volume that the working electrode could accommodate) of a GO solution prepared at a concentration of 1 mg mL^–1^ in distilled water was drop-cast onto the GC surface. The GO-modified GC was then dried in an oven at 50 °C. Subsequently, the GC/GO electrode was immersed in 0.1 mol L^–1^ PBS (pH 7), and the reduction of GO on the electrode surface was performed using the CV method. The reduction process was performed within a potential window from 0 V to −1.5 V, at a scan rate of 50 mV s^–1^, with a step potential of −2.44 mV, and a total of 20 cycles. As a result, GC/ERGO was successfully obtained.^38^

Modification of the GC/ERGO working electrode surface with 2-NP monomer (50 mmol L^–1^ in a solution of 0.1 mol L^–1^ NaOH) was performed using the CV technique in the potential range of −0.2 to 1.2 V at a scan rate of 20 mV s^–1^ (at a step voltage of 2.44 mV) in the presence of 0.1 mol L^–1^ NaNO_3_ support electrolyte for 15 cycles.^37^ At the end of this process, a GC/ERGO/P2NP sensor working electrode was prepared. The effect of the number of cycles on modification of the GC was investigated using 5, 10, 15, and 20 cycles, respectively, and the most sensitive determination for NG was performed with the working electrode prepared using 15 cycles.

The CV scans (within a potential range of −0.2 to 0.6 V at a scan rate of 50 mV s^–1^) and EIS measurements (the frequency range was set between 10 mHz and 0.1 MHz, with a resolution of 10 points per decade at 10 mV) were performed to characterize the prepared GC/ERGO/P2NP sensor electrode. Additionally, the Randles–Ševčík equation,^44^ based on the data obtained from the CV measurements, was used to calculate electrodes’ electroactive surface area. The CV scans for this calculation were carried out in a 5.0 mM [Fe­(CN)_6_]^3–/4–^ solution prepared in 0.1 M HCl with 0.1 mol L^–1^ KCl used as the supporting electrolyte. The GC/ERGO/P2NP, GC/ERGO, and bare GC electrodes’ active surface areas were determined based on this equation;

1 $$I=2.69\times 10^{5}n^{3/2}A{\;D}^{1/2}C{\;v}^{1/2}$$

where *I* denotes the peak current (μA), *A* represents the active surface area of the sensor (cm^2^), *n* corresponds to the number of electrons involved in the redox process, *D* is the diffusion coefficient (cm^2^ s^–1^), *v* specifies the scan rate (mV s^–1^), and *C* shows the concentration of analyte (mmol L^–1^).

### 2.4 Analytical Performances of the Used Electrodes

To evaluate the electroanalytical performance, a comparative study was conducted using bare GC, GC/GO, GC/ERGO, GC/P2NP, and the proposed GC/ERGO/P2NP modified electrodes. The measurements were performed using the SWV method in a 0.1 mol L^–1^ PBS (pH 7) containing 0.25 mL of ACN (total volume was 5 mL), in the presence of 20 mg L^–1^ NG. The peak currents and potentials for each electrode were recorded and analyzed using Nova 2.1.6 software to determine the electrode configuration providing the highest sensitivity.

### 2.5 Application of Electrochemical NG Determination

The prepared GC/ERGO/P2NP electrode was placed in a 5 mL measurement cell with NG in an ACN (0.25 mL) and 0.1 mol L^–1^ PBS (pH 7, 4.75 mL) mixture. The proposed method was performed within a potential range between −0.4 V and −1.8 V, using a step potential of 5 mV, an amplitude modulation of 20 mV, and a frequency of 25 Hz. The SWV method and the prepared GC/ERGO/P2NP sensor electrode were used for electrochemical determination of NG in the range of 0.5–100 mg L^–1^ (4.80 × 10^–6^–9.61 × 10^–4^ mol L^–1^). After the measurement, the characteristic reduction peak was recorded against an Ag/AgCl/3 M NaCl reference electrode. Calibration curves were constructed by using the peak current values obtained at different NG concentrations. Moreover, acetone was added to the measuring solution to evaluate the potential interference effects of nitro-aromatic and nitramine-based energetic compounds, as they are insoluble in phosphate buffer.

### 2.6 Assay in the Presence of Synthetic and Real Energetic Mixtures

The proposed SWV method was employed to analyze binary and multicomponent mixtures of synthetic and real energetic materials, each containing 5 mg L^–1^ NG in combination with TNT, DNT, RDX, Tetryl, HMX, NTO, NH_4_NO_3_, Comp. B, and Octol (100 mg L^–1^ was used for the analysis, except NTO (used 50 mg L^–1^)). Except for NH_4_NO_3_, which was dissolved in 0.1 mol L^–1^ pH 7 PB, all solutions of synthetic and real energetic materials were made at 1000 mg L^–1^ in ACN.

### 2.7 Interference Effects of Electroactive Camouflage Materials and Common Soil Ions

Several electroactive camouflage materials, such as acetylsalicylic acid, detergent, d-glucose, caffeine, and paracetamol, were investigated as potential interferent compounds because of their similarity in color and physical appearance. These materials were attempted to be dissolved in phosphate buffer (pH 7) in the proper quantities before analysis, and the solutions were sonicated in an ultrasonic bath for 30 min. After the solution was filtered through a Chromafil PET-45/25 (0.45 m filter), 0.1 mol L^–1^ pH 7 PBS was added until the flask was filled to the mark.^42^ In the presence of 200 mg L^–1^ concentrations of these materials, 20 mg L^–1^ NG was quantified with the SWV method.

In the presence of potentially interfering common soil ions, including Cl^–^, SO_4_^2–^, NO_2_^–^, NO_3_^–^, NH_4_^+^, K^+^, Na^+^, Pb^2+^, Ca^2+^, and Mg^2+^ at concentration levels of 1000 mg L^–1^, solutions were prepared containing 20 mg L^–1^ NG to evaluate the method’s selectivity. For the Cu^2+^ and Fe^3+^ ions, Lewatit S1468 (a cation exchanger resin) was used individually to prevent potential interference. For this, 100 mL of distilled water and 2 g of resin were added to an Erlenmeyer flask, and it was left to incubate for a day. The distilled water was subsequently removed by decantation. The resin was combined with NG at 100 mg L^–1^ and Fe^3+^ at 10-fold concentration levels in a total volume of 10 mL. The pH of the solution was then adjusted to 2.5. The solution’s pH was then brought down to 2.5. The sample was filtered through Chromafil PET-45/25 and transferred to a volumetric flask after being moved to a centrifuge tube and rotated for 90 min at 1000 rpm. 3.75 mL of 0.1 mol L^–1^ pH 7 PBS, 1 mL of this filtrate, and 0.25 mL of ACN were added to the cell in order to SWV measurement. To investigate the interference effect, Cu^2+^ underwent the same process.

### 2.8 SWV Method Validation versus LC–MS Method

To prepare NG-spiked clay soil samples, 1.0 g of clay soil was mixed with 2.5 mL of a 1000 mg L^–1^ NG solution, and the samples were subsequently dried at room temperature. The total solution was added in two batches of 10 mL each, followed by 5 mL of ACN, and placed in an ultrasonic bath for 5 min. The mixture was centrifuged at 5000 rpm for 5 min. The resulting supernatant was filtered through a Chromafil PET-45/25 syringe filter, collected in a 25 mL volumetric flask, and diluted to the mark (the final concentration was 100 mg L^–1^ NG). To determine NG using the devised SWV method, 0.25 mL of this solution and 4.75 mL of 0.1 mol L^–1^ (pH 7) of PB were put into the measurement cell. Sample solutions at 10–100 μg L^–1^ NG were made by diluting a 1000 mg L^–1^ NG solution in ACN for method validation against the LC–MS method.^23^ The negative ion mode electrospray ionization method was utilized for the LC–MS analysis, and the ionization voltage used was 3.5 kV. For NG (collision energy: 34.0 V), the product and precursor ions were 46.0 and 103.0 *m*/*z*, respectively. The Student’s *t*- and *F*-statistical tests were employed to validate the approach against the LC–MS determination of the NG.

## 3 Results and Discussion

### 3.1 GC/ERGO/P2NP Working Electrode Preparation

A two-step modification procedure was employed to prepare a sensor working electrode for the sensitive and selective electrochemical detection of NG in the presence of various energetic compounds. First, the surface of the GC working electrode was modified with ERGO using the CV technique. Upon examining Figure S1, the peak corresponding to the reduction of GO on the GC working electrode surface was observed at −1.37 V. Upon successive cycling, the graphene oxide on the GC electrode surface transformed into reduced graphene oxide, with a gradual decrease in signal intensity. The CV process confirmed the reduction of GO to ERGO, further supported by FTIR measurements. For these measurements, carbon-based screen-printed electrodes (SPE) were utilized, and the FTIR spectra of SPE/GO and SPE/ERGO electrodes were compared (Figure 1).

**Figure 1:**
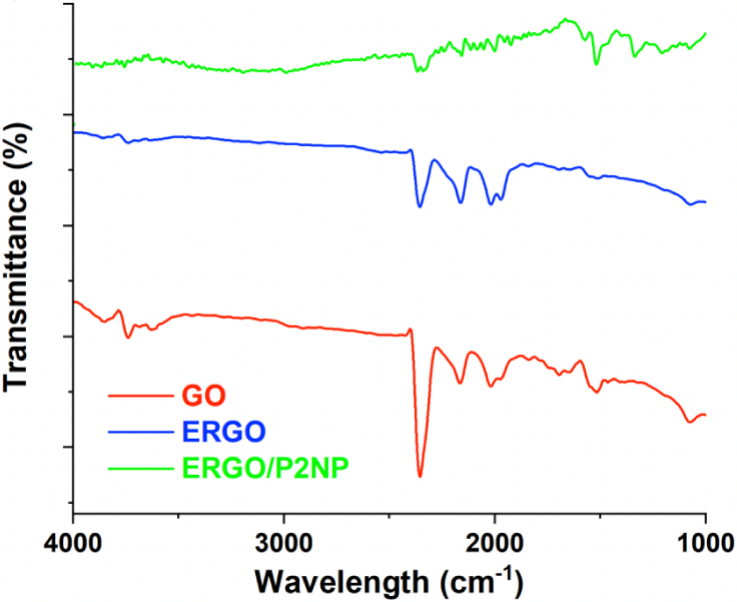
FTIR spectra of graphene oxide (GO), reduced graphene oxide (ERGO), and reduced graphene oxide/poly-2-nitrophenol (ERGO/P2NP).

Figure 1 shows the FTIR spectra of GO and ERGO. In the GO spectrum, a broad and intense band centered around 3500 cm^–1^ is observed, assigned to the stretching vibration of −OH functionalities.^45^ The strong absorption band observed at around 1720 cm^–1^ is attributed to the stretching vibrations of carbonyl (C=O) groups from carboxylic acid functionalities.^46^ Additionally, the peak near 1620 cm^–1^ can be assigned to the vibrations of aromatic C=C bonds.^47^ The absorption bands located around 1220 cm^–1^ and 1050 cm^–1^ are associated with C–O–C (epoxy) and C–O (alkoxy) stretching vibrations, respectively, revealing a GO structure rich in oxygen-containing functionalities.^48^

In contrast, in the spectrum of ERGO, the bands corresponding to oxygen-containing functional groups are seen to be considerably weakened. The O–H stretching band around 3500 cm^–1^ and the C=O stretching band at 1720 cm^–1^ are markedly diminished, suggesting the successful reduction of GO.^49^ Additionally, the peaks corresponding to epoxy and alkoxy groups in the 1000–1200 cm^–1^ region have significantly weakened. The continuation of the C=C stretching band around 1620 cm^–1^ confirms that the conjugated sp^2^ carbon structure is partially restored after the reduction process.^50^ These observations show that oxygen-containing groups are effectively removed during electrochemical reduction, resulting in a more hydrophobic and electrically conductive graphene structure.

Upon the electropolymerization of 2-nitrophenol onto the ERGO surface (ERGO/P2NP), new characteristic absorption bands emerged, confirming the successful formation of the polymer layer. Specifically, the bands in the region of 1340–1550 cm^–1^ is attributed to the symmetric and asymmetric stretching vibrations of the nitro (−NO_2_) groups present in the polymer backbone, while spectral features around 1250–1280 cm^–1^ correspond to the C–N and phenolic C–O stretching vibrations. Distinct changes in the spectral profile were observed compared to those of the pure monomer or bulk polymer. Consistent with the formation of conductive polymer/graphene composites,^51^ the characteristic peaks of the P2NP layer appeared slightly broadened and shifted. This phenomenon is supported by recent studies on phenol-adsorbed graphene derivatives^52^ and provides clear evidence for intimate interfacial interactions between the polymer and the graphene scaffold. Specifically, this spectral modulation is attributed to the strong π–π stacking interactions between the aromatic rings of poly­(2-nitrophenol) and the conjugated basal plane of ERGO, which restricts the vibrational freedom of the polymer chains.^53^

In the second step, the surface of the GC/ERGO working electrode was modified with P2NP using a 2-NP monomer solution by the CV method for 15 cycles, as described in Section 2. In Figure 2, the oxidation peak of 2-NP was observed at approximately 0.94 V using the CV technique. As the number of cycles increases, the amount of polymer deposited on the GC/ERGO working electrode surface also increases, while the monomer concentration remaining in the polymerization solution decreases. When the number of cycles exceeded 15, excessive polymer buildup resulted in a decline in electrode sensitivity. Thus, the optimal cycle count was determined to be 15 (Figure S2). 2-NP is polymerized by oxidation, forming reactive radicals that join to create a polymer film on the electrode surface using the CV method.^37^ As a result, a sensor working electrode was prepared for the determination of the NG and named the GC/ERGO/P2NP working electrode.

**Figure 2:**
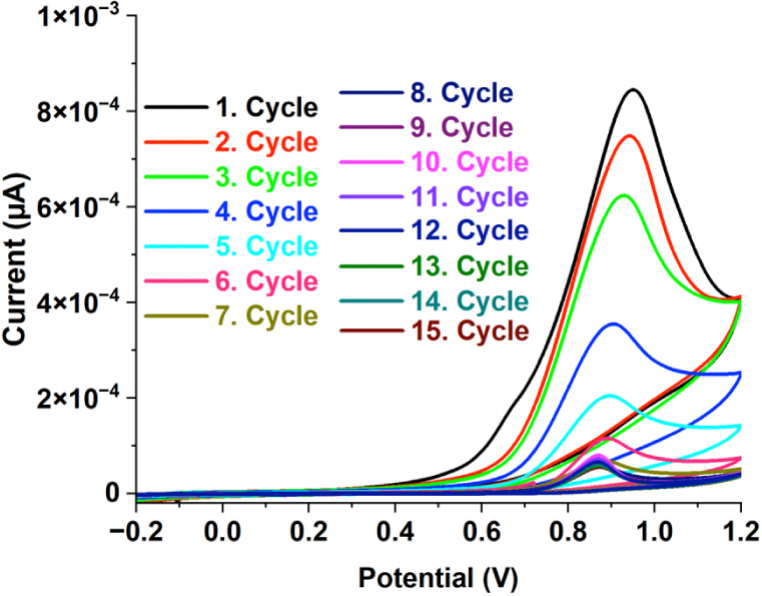
Cyclic voltammogram of the polymerization of 2-NP (50 mmol L^–1^) containing 0.1 mol L^–1^ NaNO_3_ (supporting electrolyte) (scan rate: 20 mV s^–1^).

### 3.2 Proposed Method Optimization

#### 3.2.1 Selection of the Measurement Medium and the Supporting Electrolyte

To determine the optimal measurement medium for the electrochemical analysis of NG using the prepared GC/ERGO/P2NP electrode, measurements of 20 mg L^–1^ NG were conducted in three different media: 5.0 mL of 0.1 mol L^–1^ PBS (pH 7), 0.25 mL of acetone +4.75 mL of 0.1 mol L^–1^ PBS (pH 7), and 0.5 mL of acetone +4.5 mL of 0.1 mol L^–1^ PBS (pH 7). The addition of acetone to the measurement medium was essential to facilitate the simultaneous analysis of NG with other types of energetic materials (e.g., nitroaromatics and nitramines), due to their poor water solubility and significantly higher solubility in acetone. As shown in Figure S3, the highest current response corresponding to NG’s characteristic reduction peak at −1.27 V was obtained in the medium containing 0.25 mL of acetone +4.75 mL of 0.1 mol L^–1^ PBS (pH 7). Consequently, this composition was selected for all subsequent measurements.

Additionally, various supporting electrolytes other than 0.1 mol L^–1^ PBS (pH 7, 0.1 mol L^–1^) were evaluated for the electrochemical analysis of NG using the prepared GC/ERGO/P2NP electrode. These included 0.1 mol of L^–1^ LiClO_4_, 0.5 mol of L^–1^ NaCl, 0.05 mol of L^–1^ TBABr, and 0.03 mol of L^–1^ TBABF_4_. As observed in the results presented in Figure S3, the highest current response corresponding to NG was achieved in the medium composed of 0.25 mL of acetone and 4.75 mL of phosphate buffer solution (pH 7), as previously indicated.

#### 3.2.2 Selection of the Optimal pH for Measurement

A study was conducted to determine the optimal pH of the measurement medium for the electrochemical analysis of NG by using the prepared GC/ERGO/P2NP electrode. For this purpose, 0.1 mol L^–1^ PBS with pH values of 4, 6, 7, and 8 was prepared as the supporting electrolyte, and the electrochemical measurements of 20 mg L^–1^ NG were carried out using the square wave voltammetry (SWV) technique. Figure S4 analysis revealed that the measurement medium yielding the highest current response consists of 0.25 mL of acetone and 4.75 mL of 0.1 mol L^–1^ of PBS (pH 7).

### 3.3 Analytical Performances of the Working Electrodes

The analytical performance of bare GC and modified electrodes (GC/GO, GC/ERGO, GC/P2NP, and GC/ERGO/P2NP) toward 20 mg L^–1^ NG was evaluated via the SWV method in a mixture of 0.25 mL of ACN and 4.75 mL of 0.1 M PBS (pH 7), as detailed in Section 2. In Figure 3, the reduction current was obtained as 2.18 μA for the GC electrode at −1.40 V, 1.98 μA for the GC/GO electrode at −1.05 V, 3.02 μA for the GC/ERGO electrode at −1.09 V, 5.64 μA for the GC/P2NP electrode at −1.30 V, and 6.99 μA for the GC/ERGO/P2NP modified electrode at around −1.27 V. The corrected current values were determined using Nova 2.1.6 software of the PGSTAT204. Upon evaluation of the results, the highest current value for the reduction of 20 mg L^–1^ NG was obtained using the GC/ERGO/P2NP sensor working electrode.

**Figure 3:**
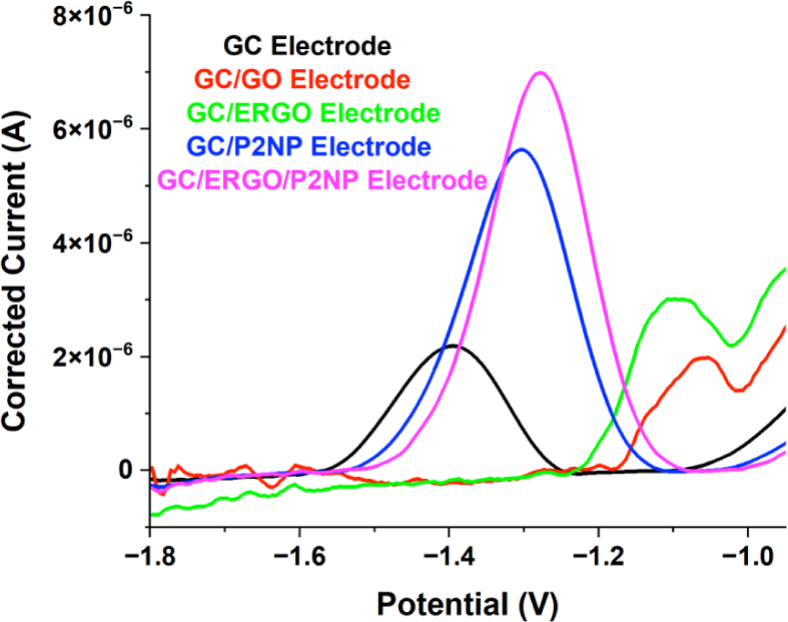
Square wave voltammograms for the reduction of 20 mg L^–1^ NG using GC, GC/GO, GC/ERGO, GC/P2NP, and GC/ERGO/P2NP working electrodes.

It is worth noting that while electropolymerization of o-nitrophenol on gold (Au) electrodes has been reported to cause electrode fouling,^54^ such a phenomenon was not observed on the GC/ERGO/P2NP electrode. This is ascribed to the compatible π–π stacking interactions between the ERGO surface and the aromatic modifier layer.^55^ Additionally, literature indicates that the presence of reduced graphene oxide creates a conductive network where nitro-aromatic groups bind effectively via hydrogen bonding and π–π interactions, preventing the formation of an insulating passivation layer.^56^ Experimentally, this is confirmed by the analytical performance data, where the reduction peak current significantly increased from 2.18 μA (bare GC) to 6.99 μA (GC/ERGO/P2NP). This 3-fold enhancement demonstrates that the composite film acts as an efficient electron transfer mediator rather than a passivation layer.

### 3.4 Electrochemical Investigation of the GC/ERGO/P2NP Modified Electrode

The CV scans and EIS measurements were performed to characterize the prepared GC/ERGO/P2NP sensor electrode. The CV scans were carried out in a 5 mmol L^–1^ [Fe­(CN)_6_]^3–/4–^ solution (5 mL, dissolved in 0.1 mol L^–1^ HCl with 0.1 mol L^–1^ KCl) using the CV method from −0.2 to 0.6 V at a 50 mV s^–1^ scan rate. The cathodic and anodic peak potentials associated with the redox couple [Fe­(CN)_6_]^3–/4–^ were obtained at around 0.31 and 0.38 V, respectively. The current values of cathodic and anodic peaks were measured as −63.78 and 72.72 μA for the GC electrode, −98.27 and 115.05 μA for the GC/ERGO electrode, and −90.94 and 108.39 μA for the GC/ERGO/P2NP modified electrode, respectively. The reduction and oxidation peak potential separation (Δ*E*_p_) was 70 mV, suggesting the electrode exhibits reversible electrochemical behavior. The obtained results confirm the electroactivity of each electrode, as illustrated in Figure S5.

The electroactive surface areas of the GC, GC/ERGO, and GC/ERGO/P2NP electrodes were calculated as 0.00243 cm^2^, 0.00375 cm^2^, and 0.00347 cm^2^, respectively, using the Randles–Ševčík equation.^44^ Upon evaluation of the obtained results, the active surface area of the GC/ERGO/P2NP working electrode increased compared to that of the GC electrode. Furthermore, studies have demonstrated that the GC/ERGO/P2NP working electrode exhibits greater sensitivity and selectivity than the GC electrode for the electrochemical determination of NG.

The EIS measurements were carried out for GC, GO/ERGO, and GC/ERGO/P2NP electrodes in the same solution used for CV measurements. The frequency range was set between 10 mHz and 0.1 MHz, with a resolution of 10 points per decade at 10 mV. The charge transfer resistance (*R*_ct_, the resistance to electron movement, was obtained by analyzing the Nyquist plots using the “electrochemical circle fit” function in Nova 2.1.6) values were 126.92 Ω for the GC electrode and 18.54 Ω for the GC/ERGO/P2NP electrode. In contrast, the GC/ERGO electrode exhibited a significantly reduced *R*_ct_ value, and no semicircle was observed. High electrical conductivity of the electrodes is shown by the low resistance values measured. When compared to the bare GC electrode in this situation, the GC/ERGO and GC/ERGO/P2NP electrodes showed noticeably greater electrical conductivity and lower charge transfer resistance^57^ (Figure 4). Additionally, the “fit and simulation” tool in Nova 2.1.6 was used to build the Randles equivalent circuit to simulate the impedance behavior for the GC, GC/ERGO, and GC/ERGO/P2NP electrodes (Figure 4). The χ^2^ values, indicating the goodness of fit for the EIS data, were calculated as 0.0296 for the GC electrode, 0.0121 for the GC/ERGO electrode, and 0.00218 for the GC/ERGO/P2NP electrode, respectively. These low χ^2^ values indicate good agreement between the recorded impedance spectra and the proposed equivalent circuit.

**Figure 4:**
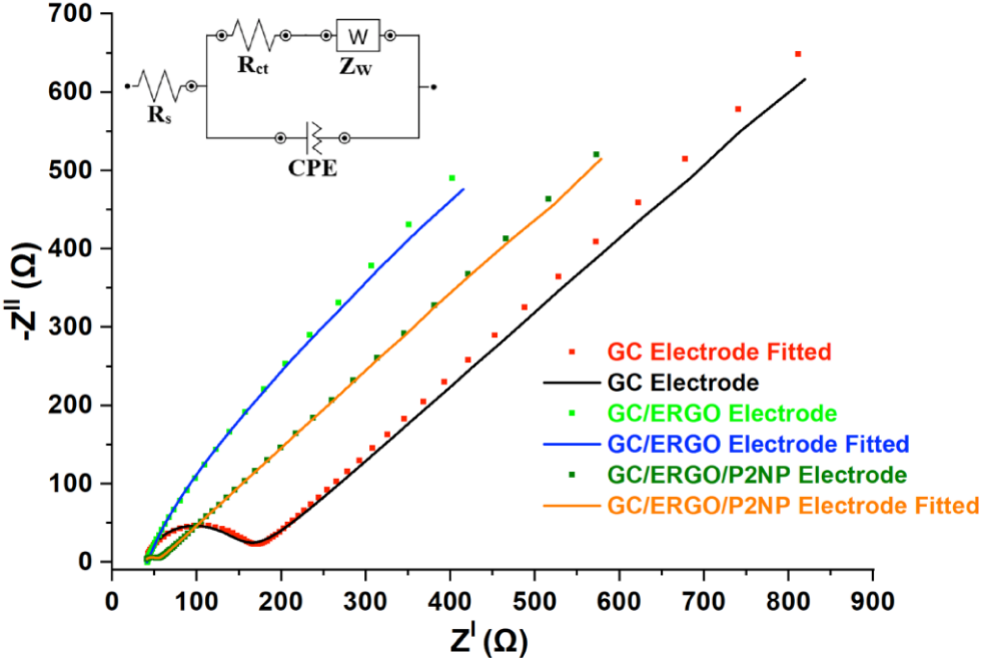
Impedance measurements of the GC, GC/ERGO, and GC/ERGO/P2NP electrodes obtained using the Potentiostat EIS method in a 0.1 mol L^–1^ HCl solution containing 5 mM [Fe­(CN)_6_]^3–/4–^ and the Randles equivalent circuit are presented as an inset figure, incorporating the constant phase element (CPE) and Warburg impedance (*Z*_w_).

### 3.5 Morphological Characterization of Electrodes

Raman spectroscopy, SEM-EDX, TEM, AFM, XRD, and XPS measurements were taken for morphological characterization of the developed electrodes.

Raman spectroscopy was applied to verify the structural evolution and surface modification of the electrode materials (Figure 5). The spectrum of pristine GO displayed an *I*_D_/*I*_G_ intensity ratio of 0.81, accompanied by a weak 2D band, characteristic of its highly disordered and oxidized structure. Upon electrochemical reduction to ERGO, the *I*_D_/*I*_G_ ratio slightly increased to 0.83. This trend is consistent with the Tuinstra-Koenig relation and indicates that the removal of oxygen-containing functional groups results in the formation of numerous smaller sp^2^ graphitic domains rather than a single continuous basal plane, thereby increasing the density of edge defects.^56,58^ Simultaneously, a broad but distinct 2D band emerged around 2700 cm^–1^, supporting partial restoration of the graphitic network and stacking order. In the case of the ERGO/P2NP composite, the *I*_D_/*I*_G_ ratio was found to be 0.84, indicating the preservation of the graphitic framework during electropolymerization. Notably, a distinct modulation of the 2D band was observed in the composite spectrum compared with that of ERGO. This phenomenon serves as strong evidence for the intimate interaction between the polymer layer and the graphene surface. As demonstrated in top-gated graphene systems, the suppression of the 2D band is attributed to the strong π–π stacking interactions and the charge-transfer-induced doping effect of the polymer coating, which dampens the second-order double resonance process characteristic of the 2D mode.^59^

**Figure 5:**
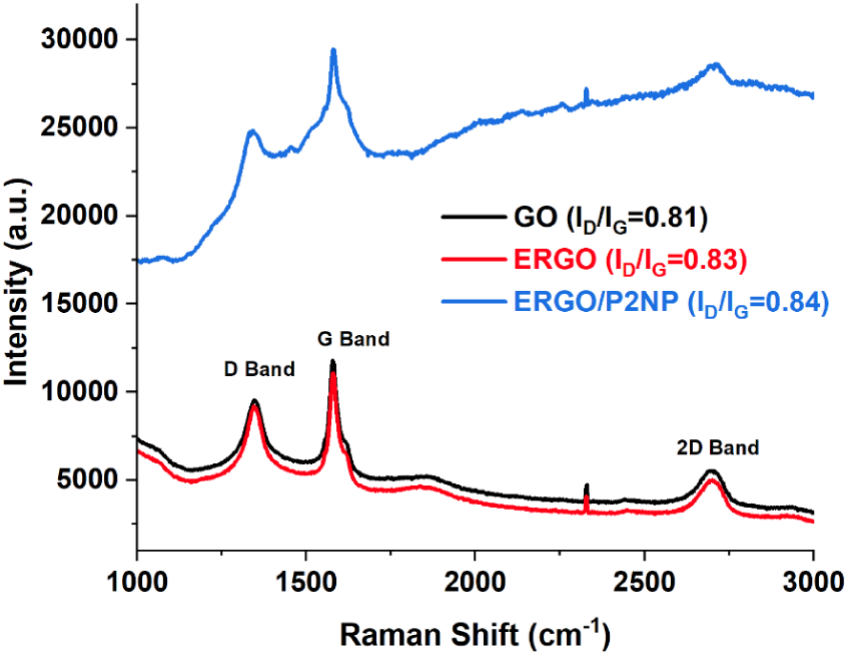
Raman spectra of GO, ERGO, and ERGO/P2NP.

The surface composition and elemental distribution of GO, ERGO, and ERGO/P2NP samples were examined by using SEM-EDX analysis. The FE-SEM image of GO (Figure 6a) reveals a typical crumpled and layered morphology associated with exfoliated graphene oxide sheets. After electrochemical reduction, ERGO (Figure 6b) exhibits a more compact and wrinkled structure, indicating partial restacking of graphene sheets owing to the elimination of oxygen-containing functional groups. For the ERGO/P2NP composite (Figure 6c), the surface morphology changes significantly, showing a relatively smoother and more homogeneous coating, which suggests successful electropolymerization of 2-nitrophenol on the ERGO surface. EDX analysis further supports these observations. The GO and ERGO samples mainly consist of carbon and oxygen, with a noticeable decrease in the oxygen content after reduction, confirming the effective conversion of GO to ERGO. For the ERGO/P2NP composite, the appearance of nitrogen in addition to carbon and oxygen originates from the polymer layer, providing clear evidence for the successful formation of the ERGO/P2NP composite electrode. Additionally, the transmission electron microscopy (TEM) measurement results of the prepared GO (1 mg mL^–1^) are given in the Supporting Information (Figure S6). These images confirm the sheet-like structure of the GO. Moreover, atomic force microscopy (AFM) analysis was performed to investigate the morphological changes during the stepwise modification of the electrode (Figure S7). AFM analysis indicates that the average roughness (*R*_a_) increased from 461.9 nm for GO to 704.5 nm for ERGO, which is attributed to the formation of a wrinkled and porous graphene network after electrochemical reduction. This trend is also consistent with the increase in RMS roughness. Subsequently, the roughness decreased to 464.8 nm for the ERGO/P2NP composite, indicating that the polymer layer partially filled the ERGO voids and resulted in a more compact and uniform surface morphology.

**Figure 6:**
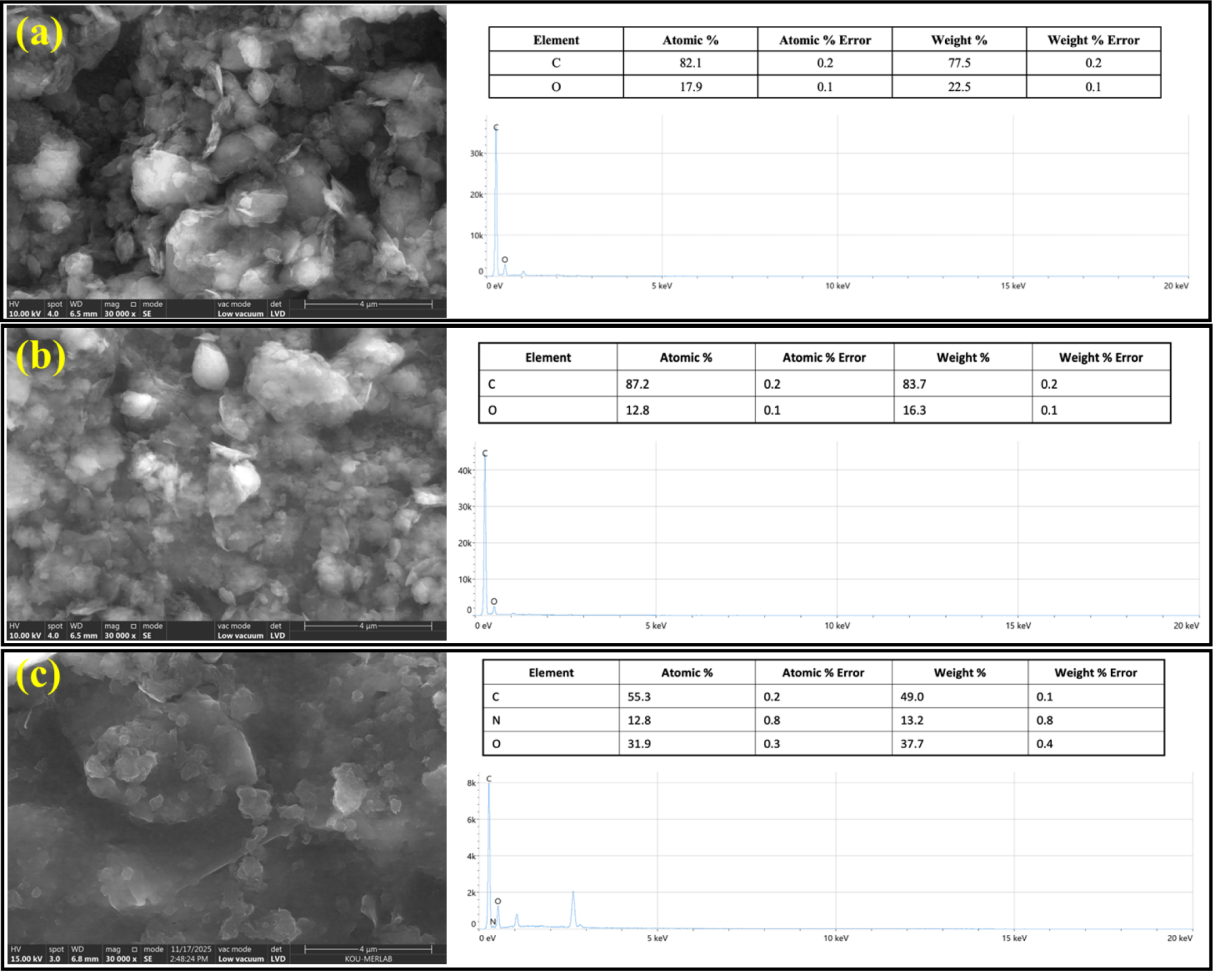
SEM images and corresponding EDX elemental analysis tables of (a) GO, (b) ERGO, and (c) ERGO/P2NP modified electrodes.

The crystallographic structures of GO, ERGO, and the ERGO/P2NP composite were investigated by XRD analysis (Figure 7). The XRD diffractogram of GO exhibited a distinct characteristic peak at 2θ ≈ 10°, corresponding to the (001) reflection (Figure 7a). This indicates an increased interlayer spacing (*d*-spacing) attributed to the intercalation of oxygenated functional groups (hydroxyl, epoxy, and carboxyl) between the graphene layers.^55,58^ In contrast, this characteristic (001) peak completely disappeared in the XRD patterns of both ERGO (Figure 7b) and the ERGO/P2NP (Figure 7c) composite. This disappearance confirms the efficient electro-reduction of GO and the removal of oxygenated groups, leading to the restoration of the graphitic structure.^58^ It is worth noting that sharp and intense diffraction peaks were observed at 2θ ≈ 26.5°, 35°, and 54° in all three samples. These peaks are attributed to the highly crystalline graphitic carbon and binder materials of the screen-printed electrode (SPE) substrate. Specifically, the prominent peak at 2θ ≈ 26.5° corresponds to the characteristic (002) plane of graphitic carbon,^60^ indicating that the modified layers were prepared as thin films. Since the X-ray penetration depth exceeded the film thickness, it resulted in dominant signals from the electrode substrate. No distinct peaks corresponding to the amorphous polymer phase were observed, likely due to its thin-film nature and the overwhelming intensity of the substrate peaks.

**Figure 7:**
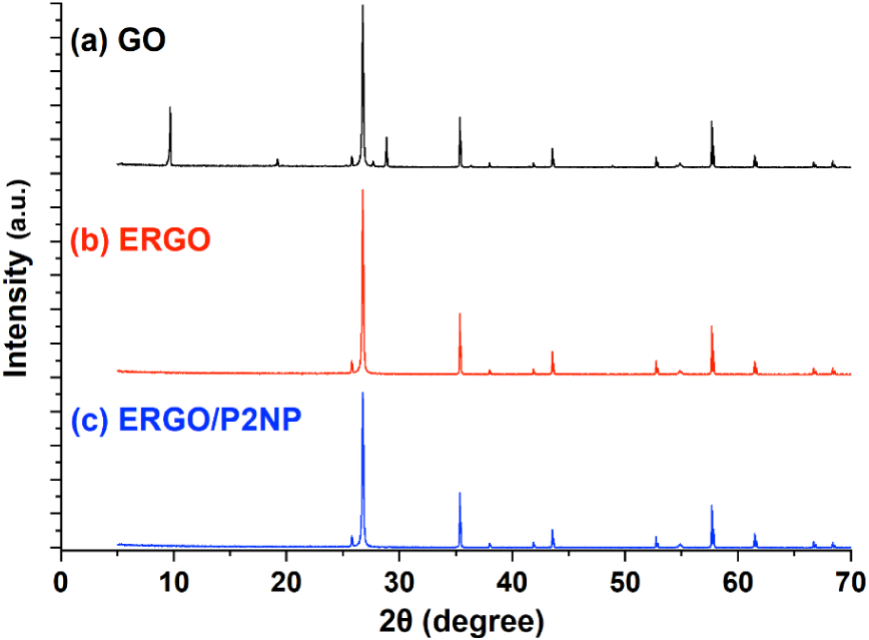
X-ray diffraction (XRD) patterns of (a) GO, (b) ERGO, and (c) ERGO/P2NP.

The stepwise modification of the sensor interface (GO, ERGO, and ERGO/P2NP) was thoroughly investigated via XPS (Figure 8). Initially, the high-resolution C 1s spectrum of the GO surface exhibited a characteristic sp^2^ C=C peak at 284.58 eV along with intense shoulders attributed to oxygenated functional groups (C–O, C=O, and O—C=O). Following electrochemical reduction (ERGO), the negative shift of the C=C peak to 284.21 eV, accompanied by a significant decrease in carbonyl/carboxyl signals, evidenced the successful removal of oxygenated groups and restoration of the conductive carbon network. In the final stage, the modification with poly­(2-nitrophenol) was confirmed by the N 1s spectrum. The emergence of fingerprint nitro (−NO_2_) peaks at 405.32 and 406.36 eV clearly indicates the presence of the nitrophenol moiety. Furthermore, a dominant peak observed at 398.81 eV is attributed to the reduced nitrogen species (amine −NH– or imine —N= linkages), which was likely generated during the electropolymerization step or due to the partial electrochemical reduction of nitro groups during scanning. Crucially, the observation of a distinct π–π shakeup satellite peak at 290.98 eV in the high-resolution C 1s spectrum verified the preservation of the conjugated aromatic system, suggesting that the stable anchoring of the polymer film onto the ERGO scaffold was primarily facilitated by strong π–π stacking interactions between the graphene basal plane and the 2-nitrophenol aromatic rings.^61–64^

**Figure 8:**
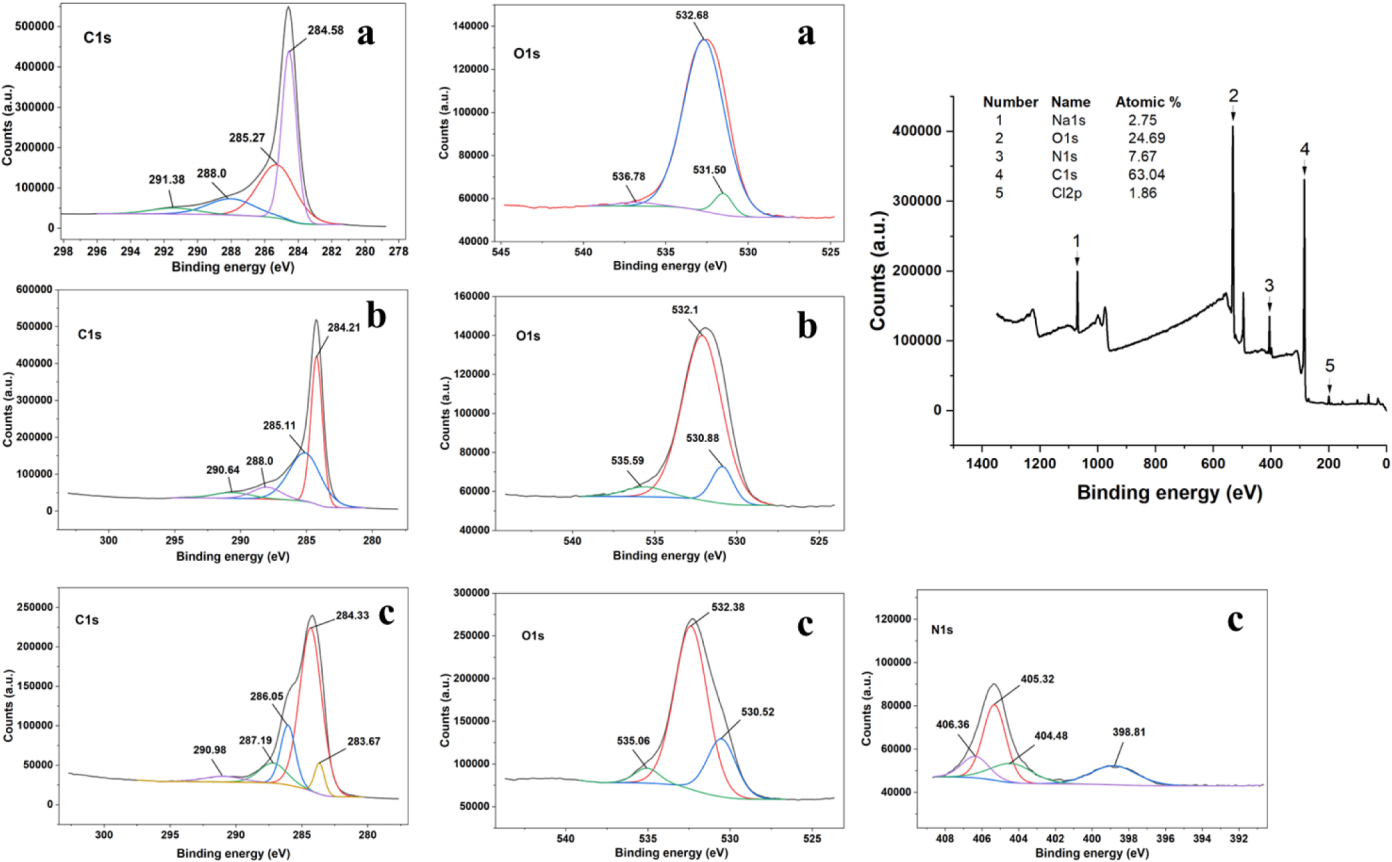
X-ray photoelectron spectroscopy (XPS) characterization of stepwise surface modification. High-resolution C 1s and O 1s spectra of (a) GO and (b) ERGO; (c) high-resolution C 1s, O 1s, and N 1s spectra along with the wide-scan survey spectrum (right panel) of the ERGO/P2NP composite.

### 3.6 The Effect of Scan Rate

To examine the scan rate effect, the CV scans were recorded from −0.4 V to −1.8 V for a solution containing 50 mg L^–1^ NG, prepared in a solvent matrix of 0.25 mL ACN and 4.75 mL 0.1 M PBS (pH 7) (Figure 9). In the experimental studies, scan rates of 10, 25, 50, 100, 200, 300, 400, and 500 mV s^–1^ were employed sequentially. The voltammograms obtained from the measurements are presented in Figure 9a. In these voltammograms, two distinct cathodic peaks are distinguishable. The first peak, observed around −0.8 V (shifting from −0.72 V), is attributed to the reduction of residual dissolved oxygen.^65^ The second and more prominent peak around −1.35 V corresponds to the reduction of NG, exhibiting a cathodic potential shift from −1.27 V with increasing scan rates, which is characteristic of irreversible electrode processes. A significant enhancement is observed in the cathodic peak current as the scan rate increases, suggesting that electroactive species are transported near the electrode surface more quickly and the kinetics of the electrochemical reaction are correspondingly accelerated. The proportional increase in current with *v* ^1/2^ confirms the consistency of the obtained results with the Randles–Ševčík equation (Figure 9b). The high correlation coefficient derived from this equation indicates that the system is diffusion-controlled. Furthermore, the slope value (0.360) obtained from the Log *I* – Log *v* plot (Figure 9c) remains below 0.5, further demonstrating that the dominant process is diffusion-controlled.^66^

**Figure 9:**
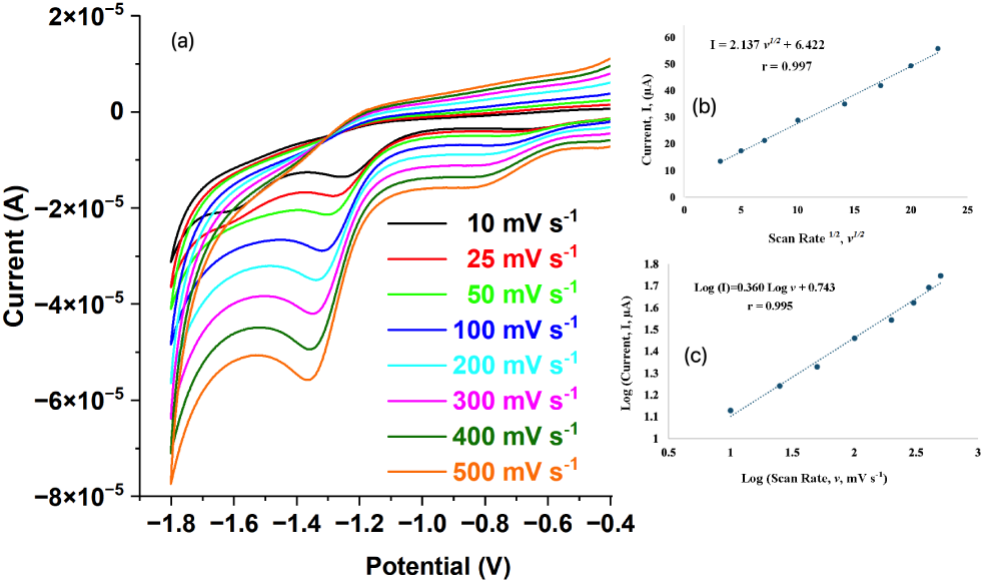
(a) Cyclic voltammograms of the GC/ERGO/P2NP electrode at various scan rates in the range from 10 to 500 mV s^–1^ in 0.25 mL of ACN and 4.75 mL of 0.1 mol L^–1^ PBS solution (pH 7) (50 mg L^–1^ NG), (b) the graph of the *I* versus *v* ^1/2^, and (c) the graph of Log *I* versus Log *v*.

The kinetic parameters were further evaluated using Laviron’s theory for irreversible electrode processes. The cathodic peak potential (*E*_pc_) and the logarithm of the scan rate (log *v*) exhibited a linear dependence defined by the Laviron equation:

2 $$E_{\mathrm{pc}}=E^{0}-\frac{2.303\;RT}{\alpha nF}\log v$$

From the slope of the *E*_pc_ versus log *v* plot (Slope = −0.0645), and assuming the transfer coefficient α is 0.5 for an irreversible process, the number of transferred electrons (*n*) was determined to be 1.83, which was approximated to 2. It should be emphasized that the value of *n* ≈ 2 obtained from the Laviron analysis represents the number of electrons involved in the rate-determining step of the electrochemical process. It is important to emphasize that while the full electrochemical reduction of NG typically follows a 6-electron/6-proton pathway (−NO_2_ → −NO → −NHOH → −NH_2_),^67^ the calculated value of *n* ≈ 2 corresponds to the rate-determining step (reduction to nitroso intermediate, −NO_2_ → −NO), which limits the kinetics observed in this analysis. Furthermore, regarding the surface coverage, the slope of the log *I* versus log *v* plot was found to be 0.360 (as shown in Figure 9c). Since this value is considerably lower than 1.0 (the theoretical value for adsorption-controlled processes) and closer to 0.5, the process is confirmed to be diffusion-controlled. Therefore, the surface coverage calculation, which is applicable to adsorption-controlled processes, was excluded in favor of the diffusion coefficient determination.

Chronoamperometry (CA) measurements were also performed to determine the diffusion coefficient (*D*) of NG. The current–time (*I*–*t* ^–1/2^) profiles were recorded for a 20 mg L^–1^ (1.92 × 10^–4^ mol L^–1^) NG solution at a step potential of −1.27 V (versus Ag/AgCl). The diffusion coefficient was calculated using the Cottrell equation:

3 $${I=nFAD}^{1/2}{C\pi }^{-1/2}{\;t}^{-1/2}$$

from the slope obtained from the Cottrell plot (*I* versus *t* ^–1/2^), which was found to be 8.0 × 10^–7^A s^1/2^, and using the concentration value of 1.92 × 10^–7^ mol cm^–3^, the *D* of NG was calculated to be 1.35 × 10^–5^ cm^2^ s^–1^ for the ERGO/P2NP modified electrode (using *n* = 6 and *A* = 0.00347 cm^2^). In addition to the diffusion coefficient, the catalytic rate constant (*k*_cat_) was calculated from chronoamperometric measurements using the Galus method by plotting *I*_NG_/*I*_B_ versus *t* ^1/2^. Using the slope value of 0.7047 and an NG concentration of 1.92 × 10^–4^ mol L^–1^, *k*_cat_ was determined to be 8.23 × 10^2^ L mol^–1^ s^–1^ indicating the efficient catalytic activity of the ERGO/P2NP modified electrode toward NG reduction.^68^

### 3.7 Electrochemical Determination of NG Using the SWV Method

The SWV method and the prepared GC/ERGO/P2NP sensor electrode were used to electrochemically determine NG within a range of 0.5–100 mg L^–1^ (4.80 × 10^–6^–9.61 × 10^–4^ mol L^–1^). Section 2.5 provides the specific experimental conditions. The detection of NG is based on the electrochemical reduction of its nitro group to an amine group. The sharp reduction peak of NG was detected at around −1.27 V as a consequence of the SWV measurement. Furthermore, the conversion of dissolved oxygen to hydrogen peroxide is responsible for the peak that can be seen in Figure 10 at −0.72 V.^65^ In addition, the calibration curve plotted using the corrected current values is presented in Figure S8. The proposed mechanism for the electro-reduction of NG to aminoguanidine (AG) was given in Figure S9.^42^

**Figure 10:**
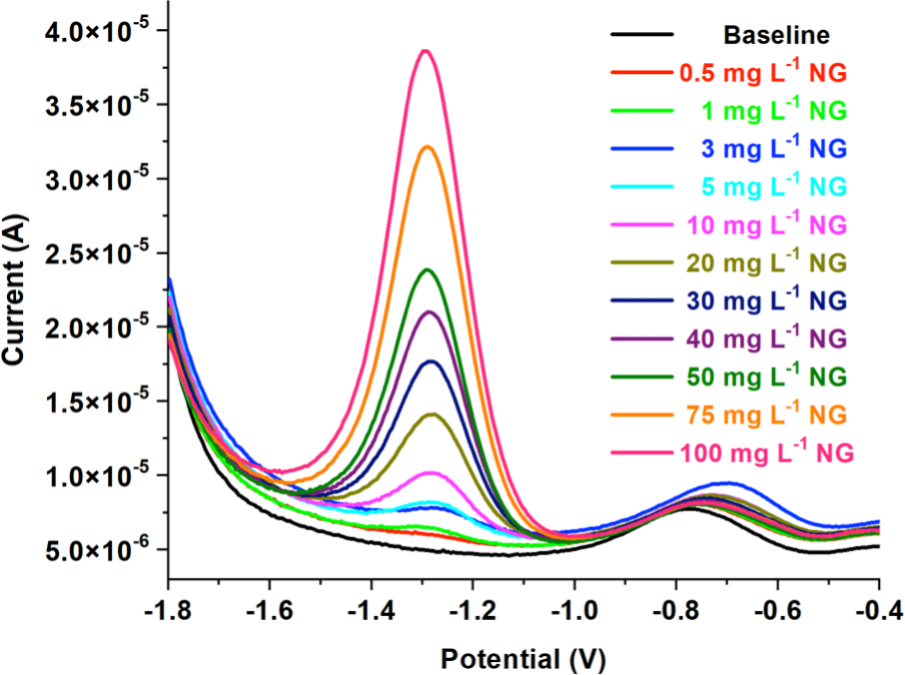
SWV voltammograms obtained from NG reduction measurements using the GC/ERGO/P2NP sensor working electrode.

The following equation describes the link between the cathodic response and NG concentration:

4 $$I_{-1.27V}\left(\mu A\right)=3.304\times 10^{-1}C_{NG}\left(mg{\;L}^{-1}\right)+6.899\;(r=0.998)$$

5 $$\Delta I_{-1.27V}\left(\mu A\right)=3.117\times 10^{-1}C_{NG}\left(mg{\;L}^{-1}\right)+0.398\;(r=0.998)$$

where *C*_NG_ is the NG concentration (mg L^–1^), *I*_–1.27 V_ is the current intensity (μA) at −1.27 V, and Δ*I*_–1.27 V_ is the corrected current intensity (μA) at −1.27 V. The relative standard deviations (RSD) for intra- and interassay measurements of NG were calculated as 2.13% and 4.63%, respectively. Additionally, the sensitivity of the GC/ERGO/P2NP electrode toward NG was calculated as 0.3304 μA L mg^–1^.

The phenolic −OH group of 2-nitrophenol and the *p*-amino (−NH_2_) groups of NG form hydrogen bonds, which are the basis of the proposed mechanism for the analyte’s enrichment on the electrode surface.^69^ Additionally, the nitro groups present in the structure of NG may also participate, to some extent, in hydrogen bonding interactions.^70^

The DFT calculations (based on B3LYP [6-31G′(d,p)]) were carried out using the GAUSSIAN-09/GAUSSVIEW-6 software (Gaussian Inc., Wallingford, USA) to better understand the binding characteristics of NG, P2NP, and NG-P2NP interaction. The energy gap (Δ*E*) between the HOMO and LUMO levels is a key parameter that determines the chemical reactivity and kinetic stability of molecules.^71,72^ As illustrated in Figure 11, the energy gap of the NG molecule was calculated to be 5.64 eV, while that of the p-2NP polymer was found to be 3.62 eV. However, upon interaction between these two molecules, the energy gap of the resulting complex decreased significantly to 0.91 eV. This substantial reduction theoretically supports the formation of a strong intermolecular interaction between NG and p-2NP, primarily attributed to hydrogen bonding.

**Figure 11:**
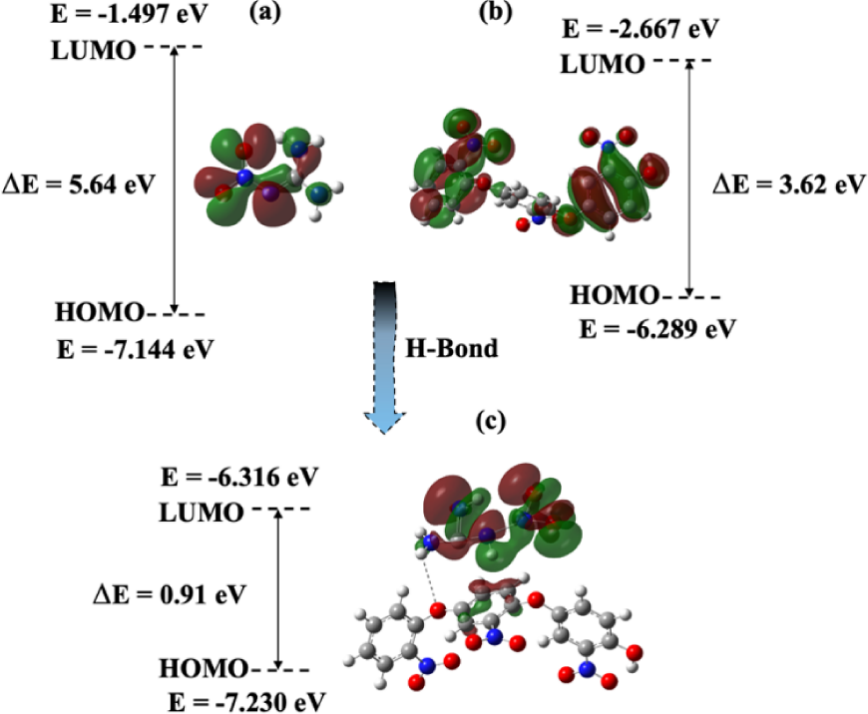
DFT computations of NG, P2NP, and NG-P2NP interaction.

### 3.8 Determination of NG in Synthetic and Real Energetic Mixtures

Five mg L^–1^ NG and 100 mg L^–1^ of different energetic compounds or their mixtures (TNT, DNT, RDX, Tetryl, HMX, NTO (used at 50 mg L^–1^), NH_4_NO_3_, Comp. B, and Octol) containing synthetic solutions were prepared for the investigation of sensitive and selective electrochemical determination of NG in synthetic and real energetic materials. Subsequently, the determination of NG was conducted utilizing the prepared GC/ERGO/P2NP sensor and the optimized SWV technique. Recoveries were determined to be between 97.8% and 105.8%. The detailed results are given in Table 1 and Figure S10.

**Table 1:** Recoveries (%) of 5 mg L^–1^ NG Containing a 20-Fold Excess of TNT, DNT, RDX, HMX, Tetryl, and Comp. B, Octol, NH_4_NO_3_, and NTO (10-Fold) (*N* = 3)

Energetic Material Mixtures	% Recovery
5 mg L^–1^ NG + 100 mg L^–1^ TNT	103.4 ± 2.1
5 mg L^–1^ NG + 100 mg L^–1^ DNT	100.8 ± 3.4
5 mg L^–1^ NG + 100 mg L^–1^ RDX	102.1 ± 2.5
5 mg L^–1^ NG + 100 mg L^–1^ HMX	104.2 ± 3.1
5 mg L^–1^ NG + 100 mg L^–1^ Tetryl	97.8 ± 2.9
5 mg L^–1^ NG + 50 mg L^–1^ NTO	105.8 ± 3.1
5 mg L^–1^ NG + 100 mg L^–1^ Comp. B	102.7 ± 2.4
5 mg L^–1^ NG + 100 mg L^–1^ Octol	102.3 ± 2.8
5 mg L^–1^ NG + 100 mg L^–1^ NH_4_NO_3_	103.9 ± 3.5

### 3.9 Investigation of the Interference Effect of Electroactive Camouflage Materials and Common Soil Ions

Electroactive substances that resemble NG in color and physical appearance (e.g., acetylsalicylic acid, aspartame, paracetamol, caffeine, d-glucose, and detergent) were evaluated for their potential interference effects on NG analysis. These substances may allow NG to be concealed, misidentified during transportation, or used as camouflage materials. Even when concentrations were ten times higher, these electroactive camouflage materials did not affect the measurement of NG (20 mg L^–1^), and the NG recovery results were 97.2%–103.4% (Figure 12).

**Figure 12:**
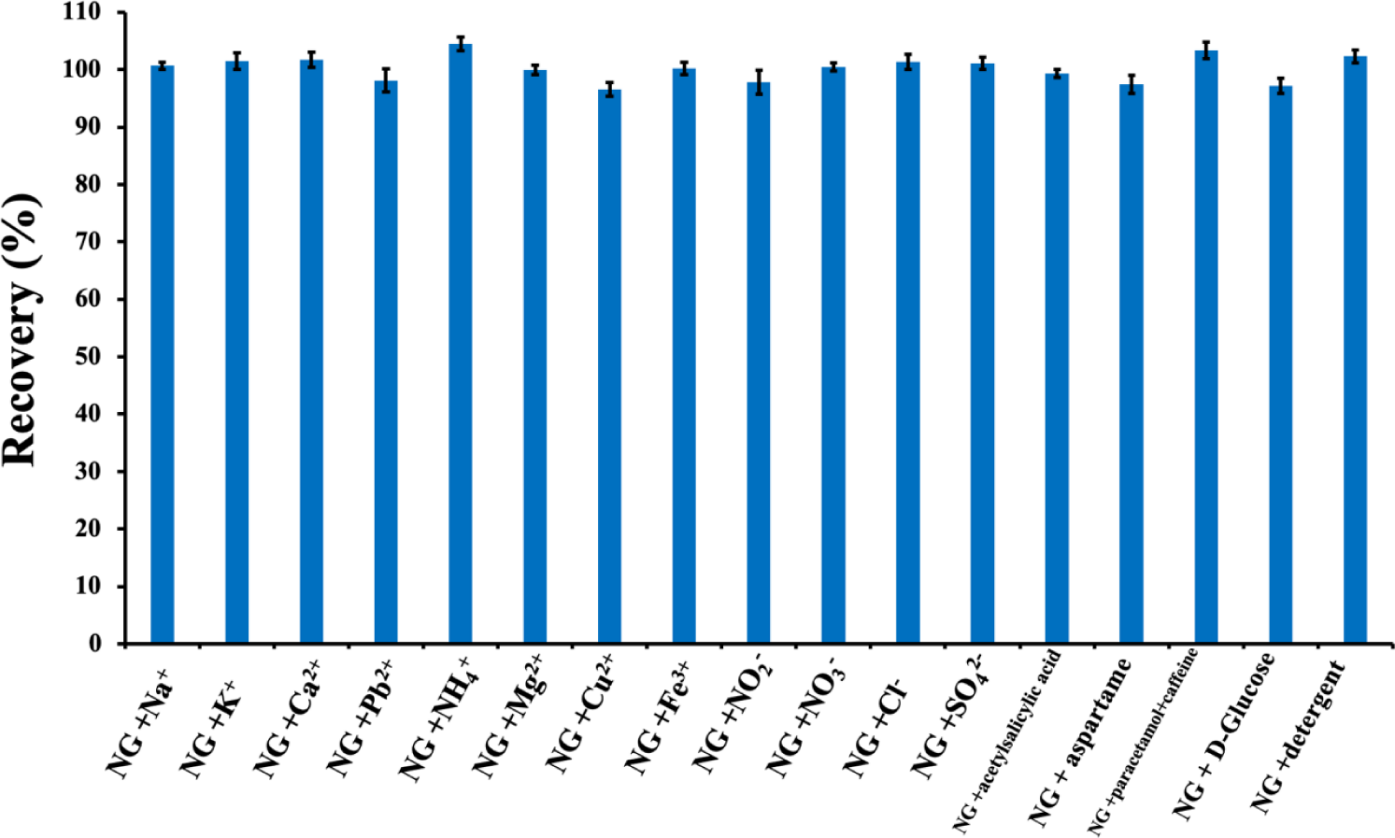
Recoveries (%) of 20 mg L^–1^ NG containing a 50-fold excess of Cl^–^, SO_4_^2–^, NO_2_^–^, NO_3_^–^, NH_4_^+^, K^+^, Na^+^, Ca^2+^, Mg^2+^, and 10-fold concentrations of Pb^2+^, Cu^2+^, Fe^3+^, acetylsalicylic acid, aspartame, paracetamol, caffeine, d-glucose, and detergent (*N* = 3).

Evaluating the possible interference effects of common soil ions, including Cl^–^, SO_4_^2–^, NO_2_^–^, NO_3_^–^, NH_4_^+^, K^+^, Na^+^, Pb^2+^, Cu^2+^, Fe^3+^, Ca^2+^, and Mg^2+^ is crucial for the highly accurate residue analysis of NG in soil. These ions were added to a 20 mg L^–1^ NG solution for this purpose at 50-fold higher levels (10-fold for Cu^2+^, Fe^3+^, and Pb^2+^), and the electrochemical detection of NG showed absolutely no interference. Although Cu^2+^ and Fe^3+^ ions initially interfered with the analysis, this was successfully eliminated using the Lewatit S1468 resin treatment described in Section 2. The measurements verified that the NG recoveries were 96.5%–104.4% (Figure 12).

### 3.10 Results of Contaminated Clay Soil Sample Analyses

A sample of contaminated clay soil was used to test the LC–MS technique for method validation.^23^ The linear calibration range for NG was investigated from 10 to 100 μg L^–1^. The calibration equation correlating the peak area to the NG concentration (*C*_NG_) was determined as

6 $$\mathrm{Peak Area}\;(\mathrm{PA})=69.91{\;C}_{\mathrm{NG}}-553.80\;(r=0.998)$$

For the LC–MS and SWV measurements, the clay soil sample contaminated with NG (containing 100 mg L^–1^ NG in the final solution) was diluted 2000 times and 20 times, respectively. Statistical evaluation of the data in Table 2 indicated no significant differences in terms of the accuracy and precision.

**Table 2:** Comparison of the Proposed SWV Technique Statistical Results with Those of the LC–MS Technique for the Detection of NG in Contaminated Clay Soil Samples

Sample/Analyte	Method	Mean Conc., mg L^–1^	Std. Dev. (σ)	S^a^	t^a^	t_table_ ^b^	F^b^	F_table_ ^b^
Contaminated Clay Soil Sample	Developed SWV Method (Voltammetric)	100.50	2.396	-	-	-	-	-
	LC–MS Method	100.01	1.635	2.051	0.385	2.306	2.148	6.39

a S^2^ = ((n_1_ – 1)­s_1_^2^ + (n_2_ – 1)­s_2_^2^)/(n_1_ + n_2_ – 2) and t = (a̅_1_ – a̅_2_)/(S­(1/n_1_ + 1/n_2_)^1/2^), where S is the pooled standard deviation, s_1_ and s_2_ are the standard deviations of the two populations with sample sizes of n_1_ and n_2_, and sample means of a̅_1_ and a̅_2_ respectively (t has (n_1_ + n_2_ – 2) degrees of freedom); here, n_1_ = n_2_ = 5.

b The comparison is based on paired data from the developed sensor and the standard reference method; for brevity, statistical outcomes are listed solely in the reference method’s row.

## 4 Conclusion

Herein, a new GC/ERGO/P2NP sensor electrode was fabricated for the electrochemical determination of NG, a water-soluble and environmentally persistent explosive compound. NG’s challenging analytical characteristics—its hydrophilic, nonvolatile, and neutral nature over a broad pH range—as well as the requirement for sensitive and specific detection techniques in environmental and security contexts, make the proposed approach significant. The sensor was fabricated in two steps: first, GO was drop-cast on the GC electrode and electrochemically reduced to ERGO and then coated with P2NP. The characterization and functionality of the sensing layer were confirmed by using CV scans, EIS, FTIR, Raman spectroscopy, SEM-EDX, TEM, AFM, XRD, and XPS measurements. The suggested mechanism for analyte enrichment on the electrode surface was the H-bonding interactions between the amino hydrogen of NG and the phenolic oxygen group of P2NP (confirmed by DFT calculations).

The fabricated sensor exhibited excellent analytical performance, with a linear detection range of 0.5–100 mg L^–1^ (4.80 × 10^–6^–9.61 × 10^–4^ mol L^–1^) and a low LOD of 0.12 mg L^–1^ (1.15 × 10^–6^ mol L^–1^). The RSD for intra-assay (repeatability) and interassay (reproducibility) measurements was found to be 2.13% and 4.63%, respectively. Furthermore, the interelectrode reproducibility was assessed by preparing three independent electrodes, yielding an RSD of 4.36%, which confirms the high reliability of the fabrication process. In terms of stability, the modified electrode preserved 95.64% of its original current response after 5 days of storage at room temperature. The sensor showed remarkable selectivity and recovery rates (96.5%–105.8%) even in complex matrices containing energetic substances, camouflage materials, and soil ions. The SWV method was successfully applied to soil samples contaminated with NG and validated against the LC–MS method using statistical *t*- and *F*-tests. Compared to the previously reported NG sensor, the GC/ERGO/P2NP-based approach showed greater analytical performance, superior sensitivity, and effective real-sample compatibility. This makes the method an effective alternative for trace-level NG detection in environmental monitoring, forensic analysis, and homeland security applications.

## Supplementary Material



## Notes

(i) Electroreduction of graphene oxide; (ii) selection of the amount of 2-NP monomer used in the electrode modification; (iii) selection of the measurement medium and the supporting electrolyte; (iv) selection of the optimal pH for measurement; (v) cyclic voltammetry (CV) scans of the GC, GC/ERGO, and GC/ERGO/P2NP electrodes; (vi) transmission electron microscopy (TEM) measurement results of the prepared GO; (vii) atomic force microscopy (AFM) measurement results of electrodes; (viii) SWV voltammograms obtained from NG measurements using the corrected current values; (ix) recovery values of NG in the presence of different types of explosive materials (PDF)

## Abbreviations

ERGO: electrochemically reduced graphene oxide
P2NP: poly-2-nitrophenol
NG: nitroguanidine
LOD: limit of detection
LC–MS: liquid chromatography–mass spectrometry
GO: graphene oxide
MWCNTs: multiwalled carbon nanotubes
PVP: polyvinylpyrrolidone
CV: cyclic voltammetry
EIS: electrochemical impedance spectroscopy
SWV: square-wave voltammetry
TNT: 2,4,6-trinitrotoluene
DNT: 2,4-dinitrotoluene
Tetryl: 2,4,6-trinitrophenylmethylnitramine
RDX: 1,3,5-trinitro-1,3,5-triazacyclohexane
HMX: octahydro-1,3,5,7-tetranitro-1,3,5,7-tetrazosine
NTO: 3-nitro-1,2,4-triazol-5-one
PETN: 3-(nitrooxy)­nitrate-2,2-bis­[(nitrooxy)­methyl]­propyl
TBABr: tetrabutylammonium bromide
TBATBF_4_: tetrabutylammonium tetrafluoroborate
GC: glassy carbon
ACN: aceton
DFT: density functional theory
FTIR: Fourier transform infrared spectrometer
SEM-EDX: scanning electron microscopy with energy-dispersive X-ray spectroscopy
XRD: X-ray diffraction
XPS: X-ray photoelectron spectroscopy
TEM: transmission electron microscopy
AFM: atomic force microscopy
