# Supplementary material for: Electrochemical Determination of Nitroguanidine among Other Explosives Using Polymerized 2‑Nitrophenol/Reduced Graphene Oxide-Modified Glassy Carbon Electrode
Source: ACS Omega. 2026 Feb 20;11(9):14760–73. doi: 10.1021/acsomega.5c10881 (PMC12980411; doi:10.1021/acsomega.5c10881)
Supplement: Supplementary file 1 [file ao5c10881_si_001.pdf]

SUPPORTING INFORMATION

Electrochemical Determination of Nitroguanidine Among Other Explosives Using Polymerized 2-Nitrophenol/Reduced Graphene Oxide-Modified Glassy Carbon Electrode

Şener SAĞLAM*

*Engineering Faculty, Chemistry Department, Istanbul University−Cerrahpaşa, 34320, Avcılar, Istanbul, Türkiye

*Corresponding Author: sener.saglam@iuc.edu.tr

Supporting information contains; (i) Electroreduction of Graphene Oxide, (ii) Selection of the Amount of 2-NP Monomer Used in the Electrode Modification, (iii) Selection of the Measurement Medium and the Supporting Electrolyte, (iv) Selection of the Optimal pH for Measurement, (v) Cyclic Voltammety (CV) Scans of the GC, GC/ERGO, and GC/ERGO/P2NP Electrodes, (vi) Transmission Electron Microscopy (TEM) Measurement Results of the Prepared GO, (vii) Atomic Force Microscopy (AFM) Measurement Results of Electrodes, (viii) SWV Voltammograms Obtained from NG Measurements Using the Corrected Current Values, (ix) Recovery Values of NG in the Presence of Different Type Explosive Materials

(i) Electroreduction of Graphene Oxide

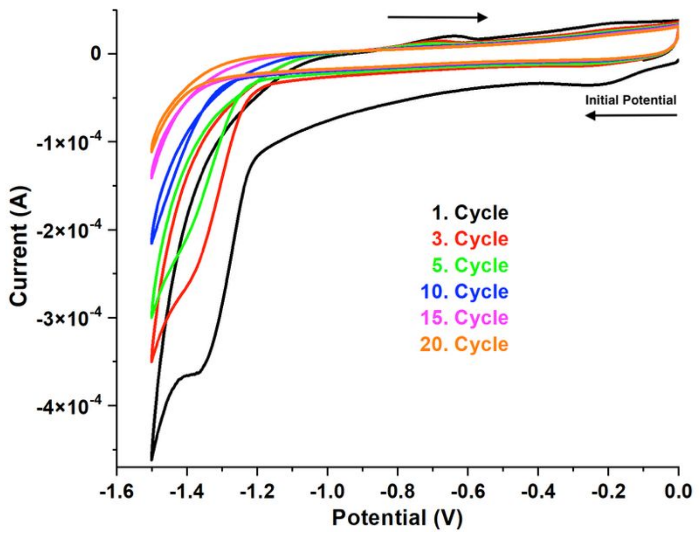

Figure S1: Cyclic voltammograms of electro-reduction of graphene oxide on the glassy carbon working electrode surface.

(ii) Selection of the Amount of 2-NP Monomer Used in the Electrode Modification

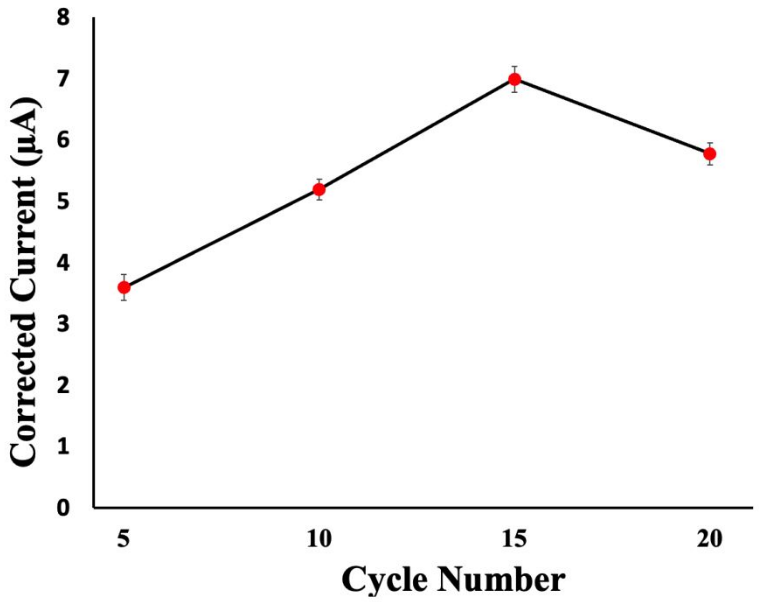

Figure S2: Effect of polymerization cycle number on the corrected current response of the GC/ERGO/P2NP modified electrode towards 20 mg L^{-1} NG using the SWV method.

(iii) Selection of the Measurement Medium and the Supporting Electrolyte

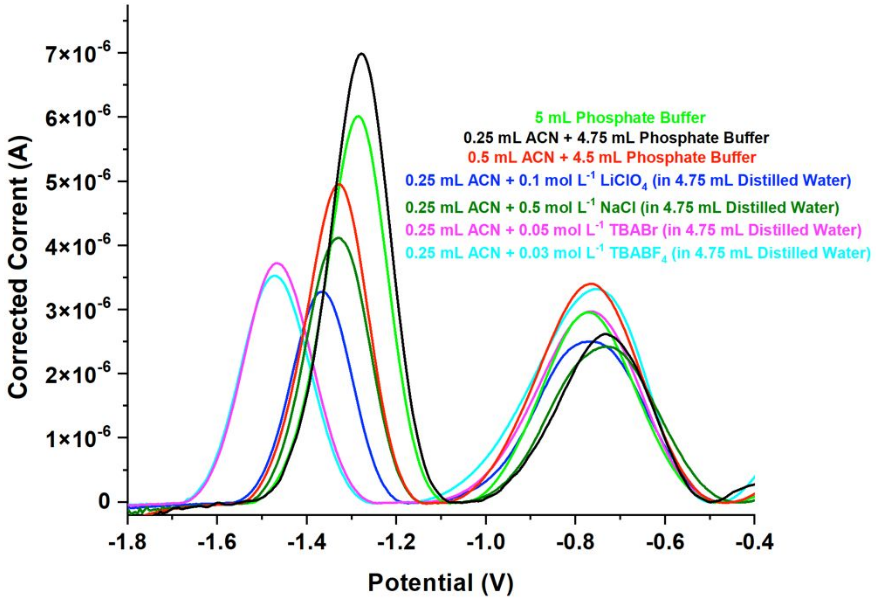

Figure S3: The square wave voltammograms of 20 mg L^{-1} NG in different solution mixtures medium.

(iv) Selection of the Optimal pH for Measurement

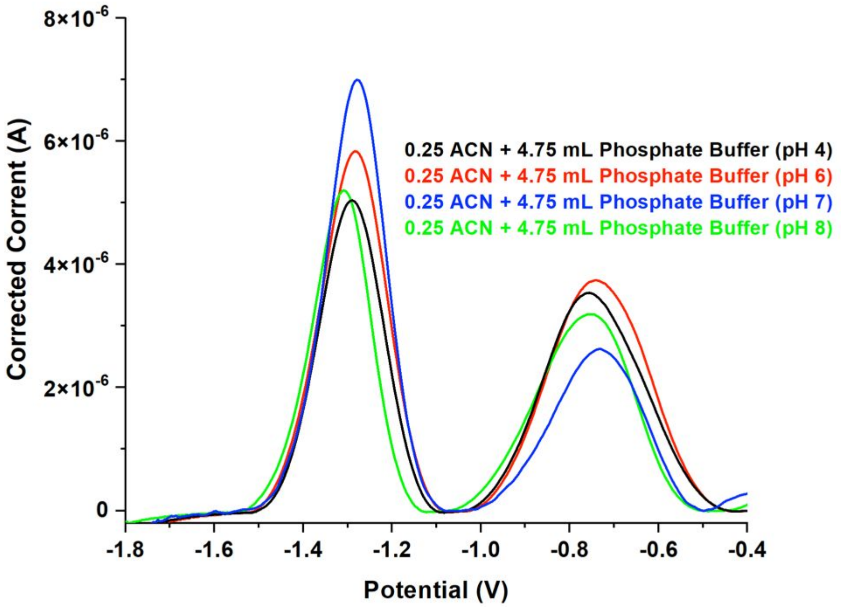

Figure S4: The square wave voltammograms of 20 mg L^{-1} NG in different pH medium.

(v) Cyclic Voltammety (CV) Scans of the GC, GC/ERGO, and GC/ERGO/P2NP Electrodes

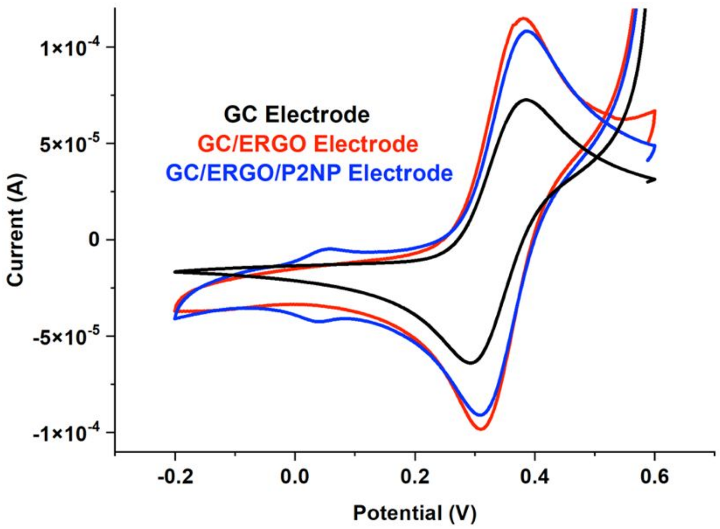

Figure S5: The cyclic voltammograms of GC, GC/ERGO, and GC/ERGO/P2NP were recorded in a 5.0 mmol L^{-1} $[Fe(CN)_6]^{3-/4-}$ solution, prepared with 0.10 mol L^{-1} KCl and 0.10 mol L^{-1} HCl as supporting electrolytes.

(vi) Transmission Electron Microscopy (TEM) measurement results of the prepared GO

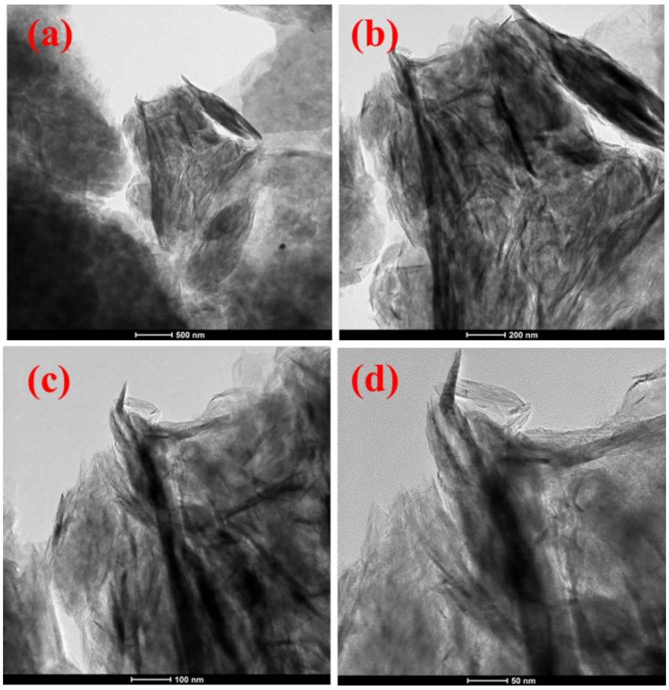

Figure S6: Transmission electron microscopy (TEM) images of the graphene oxide (GO) sheets at different magnifications.

(vii) Atomic Force Microscopy (AFM) Measurement Results of Electrodes

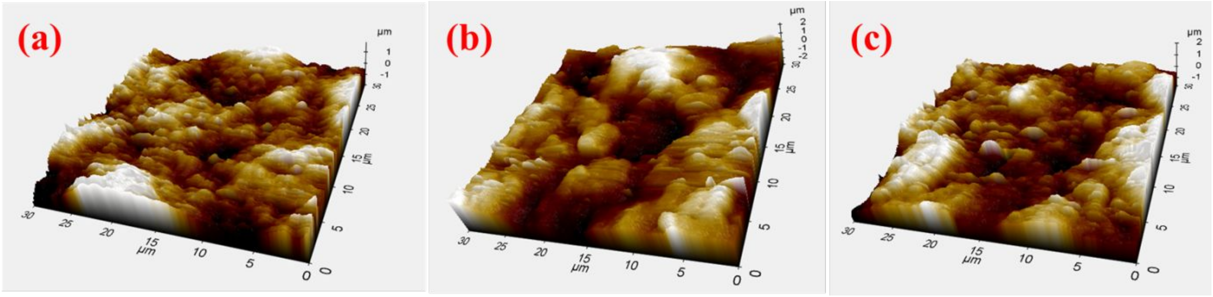

Figure S7: Three-dimensional AFM images of GO (a), ERGO (b), and ERGO/P2NP.

(viii) SWV Voltammograms Obtained from NG Measurements Using the Corrected Current Values

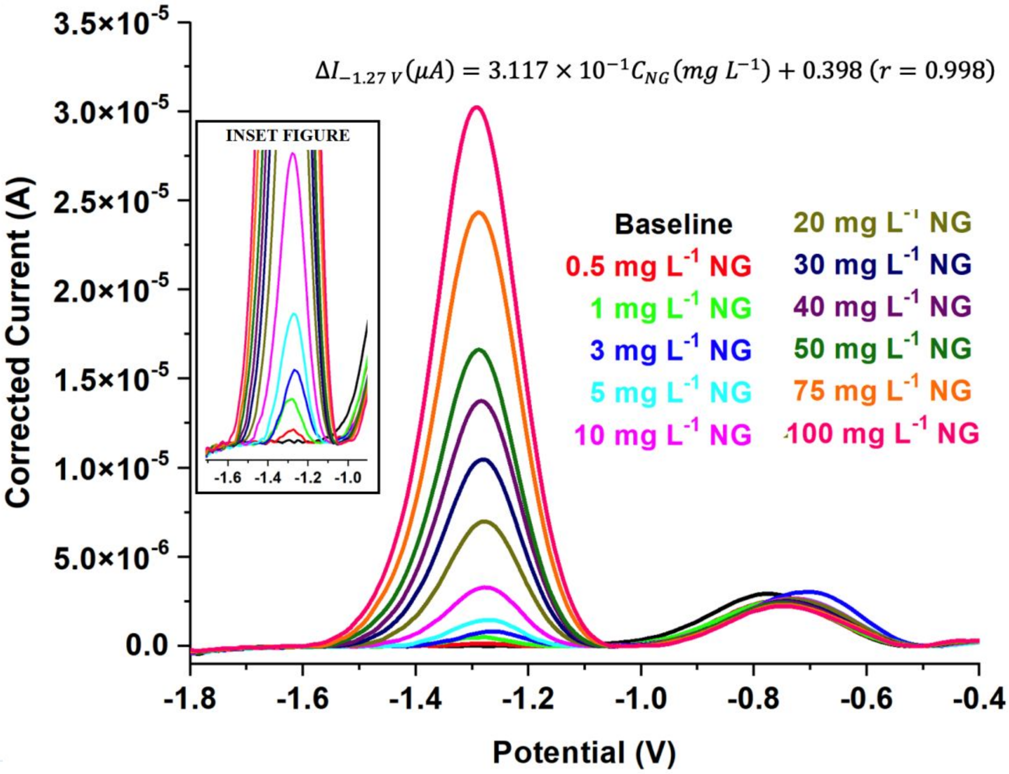

Figure S8: The SWV voltammograms obtained from NG measurements (constructed with the corrected current values) using the GC/ERGO/P2NP sensor working electrode.

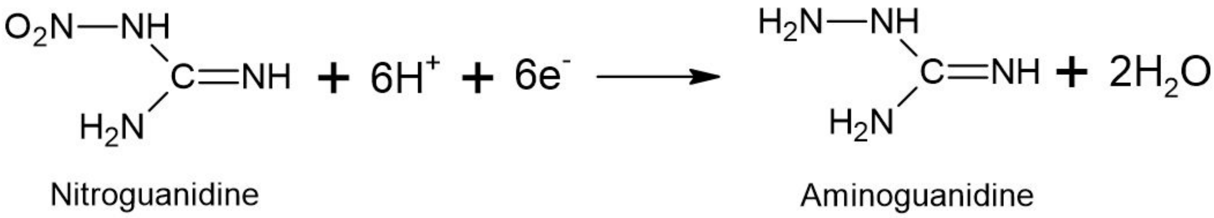

Figure S9: The proposed mechanism for the electrochemical reduction of Nitroguanidine (NG) to Aminoguanidine (AG) [1].

(ix) Recovery Values of NG in the Presence of Different Type Explosive Materials

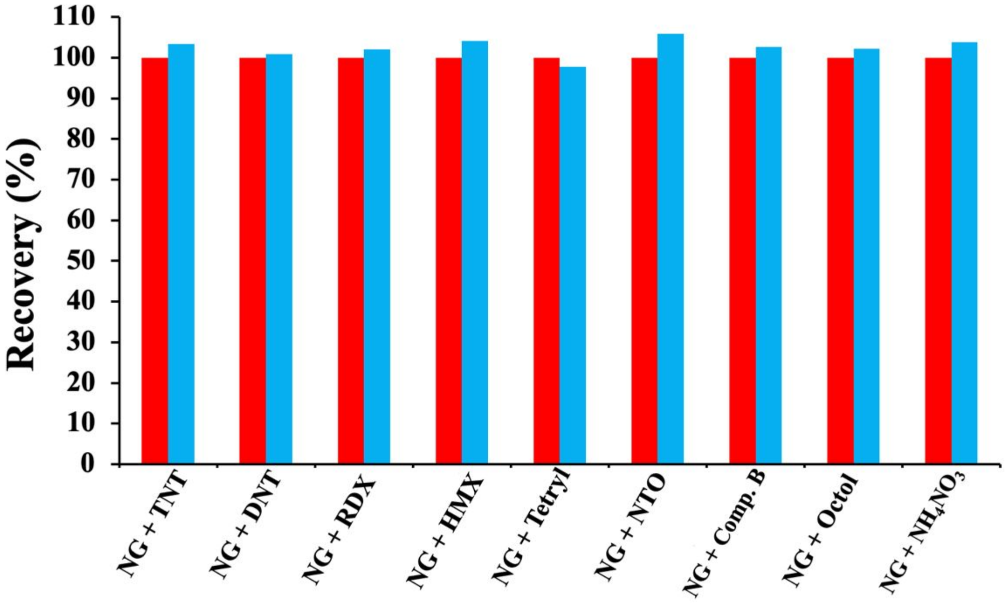

Figure S10: Recovery (%) values of 5 mg L^{-1} NG in the presence of energetic material mixtures.

Reference

(1) Alassane Moussa, A. K.; Sağlam, Ş.; Üzer, A.; Apak, R. A Novel Electrochemical Sensor for Nitroguanidine Determination Using a Glassy Carbon Electrode Modified with Multi-Walled Carbon Nanotubes and Polyvinylpyrrolidone. *New Journal of Chemistry* **2022**, *46* (21), 10081–10088. https://doi.org/10.1039/d2nj00697a.
